# Framework for an effective assessment: From rocky roads to silk route

**DOI:** 10.12669/pjms.332.12334

**Published:** 2017

**Authors:** Zarrin Seema Siddiqui

**Affiliations:** 1 Dr. Zarrin Seema Siddiqui, PhD. Education Centre, The University of Western Australia, Perth, Australia

**Keywords:** Assessment, Health professions, Medical Education

## Abstract

A defensible assessment is the cornerstone of health professions education and requires a careful approach from all the stakeholders involved in designing and delivering the curriculum. In this article, a framework is described using four essential attributes that define an effective assessment. These attributes include orientation and clarification of expectations for both faculty and students followed by a well-structured learning experience and constant evaluation of the assessment program.

## BACKGROUND

### Effective assessment doesn’t just happen. It emerges over time as an outcome of thoughtful planning….^1^

Assessment is the cornerstone of educational cycle because it is the design of an assessment system that influences learning,^2^ and therefore has been a constant focus of educational research. As far as health professionals are concerned, the educators are not only required to certify that a person should be awarded a qualification according to institution’s legislations but they also have got responsibility to ensure that the certified health professionals are fit to deliver appropriate healthcare to the community. This implies that the assessors may need to defend their decisions if a legal challenge occurs or an accreditation committee questions the assessment process. There is evidence that students identified as borderline of pass/fail decisions may graduate and cause concern regarding their competence.^3^ In another study, over thirty percent of assessors identified their reluctance to fail a student.^4^ Literature therefore recommends that assessment needs to be effective and that it is informed and governed by a set of principles which include alignment with the learning outcomes, fitness for the purpose of assessment, an assessment that is reliable, valid constant, fair transparent, inclusive and sustainable.^5^

**Fig.1:**
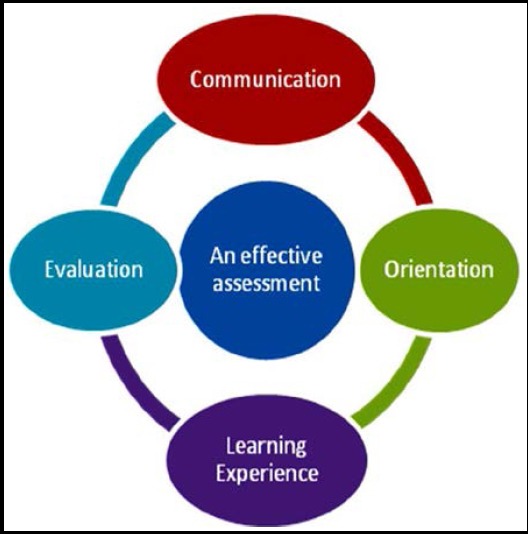
COLE framework for an effective assessment.

In this article a framework for an effective assessment is proposed (Fig.1) with four essential attributes which include communication about the assessment, orientation to the assessment, embedding assessment within the learning experience and evaluation of the assessment. Each of these attributes is discussed in detail using examples from the author’s personal experience and the literature.

### Communication

A clear communication between academics and trainees regarding the assessment requirements is the key to an effective assessment. For students it is important that they know what the assessment requirements are, what are the deadlines and what is the criteria used. If there is any scaling of marks required or standard setting mechanisms applied, the details should be documented and available to students, staff and external reviewers.

One good example is the use of progress testing where students are assessed against the outcomes that are expected at graduate level.^6^ Students need to know why it is required in order to minimise the anxiety associated with assessment. The same holds true for the use of portfolios where medical students found the independent learning required to construct a portfolio as threatening given there was so much focus on knowledge acquisition.^7^ The assessment policy of the faculty should also be made available to students and staff so the requirements to progress within the course are known to all.

### Orientation

Besides communicating the expectations and requirements for assessment, orientation to a particular assessment is another crucial factor and this applies to both staff and students. There has been evidence in the literature that staff in clinical setting did not understand the assessment forms created by the educators as the terminology was not familiar to the clinical supervisors.^8^ They may interpret items on a form differently and may use diverse criteria for assigned ratings.^9^ Assessors must be fully conversant and trained with assessment methods used by the Faculty. For example, Vassiliou et al. introduced a new tool for evaluation of intraoperative laparoscopic skills.^10^ The authors ensured that all of the assessors observing the performance of trainees have had experience with laparoscopic surgery and were familiar with the steps involved in the procedure. The observers were further trained by providing detailed instructions and reviewing the items on the checklist which resulted in an excellent internal consistency in terms of reliability. One study investigating perceptions of mentors regarding use of portfolios has suggested that if portfolios are used to promote self-directed learning skills among students, their mentors should be introduced to the relevant literature and develop their own self-directed learning skills. They have further added that mentors should experience the portfolio process themselves as learners.^11^ Lennie and Juwah have recommended that faculty development in assessment be offered in areas such as pedagogy, range of activities, standardization, moderation of assessment activities, grading and feedback/feed forward after assessment.

Students also require orientation towards new assessment tools. For example, a student may have never experienced an Objective Structured Clinical Examination (OSCE) or oral examinations in their academic setting. This can provoke anxiety among students and affect the outcome of the assessment. Faculty should make provision in time table to acquaint students with assessment tools and grading criteria early in the course to minimize any stress. Use of formative assessment using the same tools will not only provide student with feedback but will also let them experience the assessment tool itself.

### Learning Experience

Assessment is now seen in terms of educational effectiveness and itself as a learning experience which provides the learners with tasks that are authentic, simulating what they are expected to perform in real life. This has led to introduction of formative assessments commonly known as assessment for learning, workplace based assessments where trainee has opportunity to be directly observed and receive feedback on his performance so he/she can improve on subsequent assessments.^12^

With the advent of technology the e-assessments and simulation based assessments are gaining popularity which is helpful to student as they provide instant feedback and have empowered students to be more responsible for their learning. There has been introduction of tools like portfolio based assessment where a holistic picture of the trainees is assessed for fitness to practice. Some institutions are also offering Capstone experiences providing students opportunities to look beyond the medical model of practice.^13^

### Evaluation

Like all educational interventions and programs, evaluation of assessment is also required to assess if it is serving the purpose and is defensible. The characteristics that one looks for in the evaluation of an assessment are best defined in terms of the Utility formula and include estimation of reliability, validity, practicality, standardisation and educational effectiveness using quantitative and qualitative measures.^14^

### Reliability

Reliability of a test is a measure to produce similar results when applied at two different points in time.^15^ Let’s say Huma administers a test to her students in Biochemistry. If Huma administers the same test to her students a second time, one would expect consistency in the scores received in two tests for each student.

Assessing a test’s reliability is typically easier than assessing its validity. Statistical methods are used to measure the consistency within test results such as Alpha Coefficient or Kuder Richardson 21. Recently generalizability theory and D-study is also used for example Wilkinson et al used the same to report that for adequate reliability of DOPS, a trainee needs to be observed by a minimum of three assessors each observing a minimum of two procedures.^16^ Alves de Lima found that to achieve a reliable assessment using Mini CEX an approximate sample of nine encounters was required each with a different assessor, four encounters with two observers per encounter and three encounters when three observers are used.^17^

In performance based assessments, Objective Structured Clinical Examination (OSCE) has gained importance because of its reliability and the reason behind it is the predetermined criteria for each station, the sampling of clinical skills and cases that portray a more reliable assessment of a trainee and multiple assessors who are independently assessing at different stations minimising bias.^18^

Simple measures such as correlations between two scores given by a scorer at different times or correlations between two scorers can also be used to measure the reliability of an assessment. For example Ahsan makes a video recording of each of his dental students performing a high fidelity simulation. He grades each of the videos and then a month later, he examines the video recordings again and re-scores them. He then computes a correlation between two sets of grades to see the correlation between his scores on for each student on two separate occasions. This is an example of intra-rater reliability.

On the other hand another example from a recent study is where authors developed a history and physical assessment form and used two paediatrician evaluators to assess ten student write-ups. They observed that the inter-rater reliability was higher for each item on the form compared to the overall assessment score.^19^

### Validity

Validity requires evidence from different sources that the assessment is measuring its intended purpose.^20^ An assessment is not valid if it is assessing the professionalism using a written assessment because professionalism involves attributes that need to be observed in practice by multiple observers using multiple observations. Therefore structured or open ended questionnaires are used by members of healthcare team to inform 360 degree appraisal for specialist registrars.^21^ Likewise if standardised patients are exclusively used to assess the clinical competence of a trainee, it will not be a valid assessment of competence as the trainee will not receive timely feedback from the faculty as a result of direct observation of his/her practice.^22^

There are different types of validity which are described as under.

***a. Face validity***: an assessment is considered to have face validity if a number of judges ranging from experts to trainees agree that it is measuring what it intends to measure.
***b. Content validity***: The content validity of an assessment takes into consideration both the curricular content and the expectations of the learners at various stages of their training in order to ensure that there is congruency in the learning outcomes and assessments offered to the students.^23^ The process is often referred to blueprinting where faculty maps the content of the assessment against the learning outcomes using a matrix. External examiners can also retrospectively comment on the content validity of an assessment.
***c. Construct validity***: Construct validity refers that the assessment is measuring the construct that it is intended to measure. An analysis of in-training examination for residents in radiation oncology demonstrated its construct validity as the performance of residents improved with each year of additional oncology training but it was also observed that this effect persists only until level three of biology and physics training.^24^
***d. Concurrent validity***: is the extent to which an assessment correlates with other test of the same construct.^25^ In a recent review, Mini Clinical Exercises used in the training of medical graduates have been found to have concurrent validity with the other tools measuring the clinical performance.^26^
***e. Predictive validity***: is determined to assess if the said assessment can predict the future performance of the students. In a study measuring clinical performance by successfully completed clinical procedures in dentistry, it was observed that this measure has moderate positive correlation with performance in an OSCE.^27^ Another study from UK demonstrated that the selection process used for General Practice training predict their future performance on MRCGP examination.^28^
***f. Practicality/Feasibility***: A test which is highly valid yet too time consuming to administer or score, costly to purchase and is not usable is less likely to be acceptable by stakeholders. Among workplace based assessments, Direct Observation of Procedural Skills (DOPS) is considered a feasible examination as it requires only one assessor,^29^ yet there is another study which considered feasibility as a problem as DOPS forms were completed and returned by only 33% of trainees in their study.^30^ The same problem may also occur when not all the relevant procedures are encountered by a trainee in practice so they cannot be observed directly by an assessor.

Another example is the entry to postgraduate examination at the College of Physicians & Surgeons Pakistan in its formative years. The assessment required a written examination followed by a viva with four assessors. In the initial phase it was manageable but with the increasing number of applicants it became logistically difficult to manage and the assessment was changed to two written assessments comprising knowledge of basic sciences held at same day at various centres throughout the country.

One other illustration is of a medical course where Objective Structured Clinical Examination (OSCE) was held four times during the six year program. While it was considered a reliable exam, it was too time consuming and costly in terms of both physical and human resources hence the curriculum committee decided to reduce the number of OSCEs to two, with introduction of another assessment tool and more robust measures undertaken towards faculty training in development and administration so the assessment still maintains appropriate reliability and validity. One may argue that if OSCE is so costly then why not get rid of it altogether? The right answer will be that it is the cost effectiveness that compels faculty to persist with an OSCE because it clearly enables them to discriminate between different levels of performance of trainees.

At an individual level, consider example of Sonia who wants to measure clinical reasoning of her trainees. She considers developing a test where each student would select a case from their logbook to discuss in detail with Sonia in a one on one interview. Sonia feels that this test will have a higher content validity as she can assess the depth of knowledge. However as it will be too time consuming for her to administer she decided against it. Later she found that there is a commercial organisation that offers a test bank to be used which can assess the same however it would cost about 10$ per student which is again not practical. Finally, Sonia developed her own written examination which comprises case based scenarios where students have to justify their responses as well as four structured short answer questions which was a more practical approach.

Therefore when an assessment is evaluated, the feasibility in terms of cost and acceptability is also considered.

### Educational Effectiveness

Feedback from students and faculty is also required to assess the educational effectiveness/impact of an assessment. There is evidence that students rely on what is covered in the tests/assessment for the purpose of what they need to learn. Usually these are part of the overall course evaluations. These should be embedded within the quality assurance processes looking explicitly at the assessment and be considered in making decisions. At the author’s institution student representation is ensured in all committees looking at various aspects of curriculum so their feedback is continuously available and is acted upon as and when required.

## CONCLUSION

An effective assessment is crucial to health professions education as this discipline has got the obligation to provide community with health professionals who are safe and competent practitioners. A framework has been presented which includes all the attributes that contribute to an effective assessment, i.e. the expectations are communicated in a timely way with appropriate orientation to both students and staff and is embedded within learning experience with opportunities for feedback with constant evaluation of the process and product. Yet it should be kept in mind that in real world an educator is always faced with problem as to how they maintain the adequate balance between different constructs and a compromise is inevitable depending on the purpose of assessment and the regulations of the institutions.
